# Sale of Diplomas

**Published:** 1880-04

**Authors:** 


### ARTICLE II.

### *Sale of Diplomas.*

The following communication has been received from the Bureau of Education, and we trust it will receive the attention its importance demands, and this outrage be summarily stopped:

DEPARTMENT OF THE INTERIOR,
BUREAU OF EDUCATION,
*Washington, D. C., March* 26, 1880.

DEAR SIR: I have the honor to invite your attention to the following important letter from the United States Minister at Berlin, of the 2d ultimo, and to the communication from the Honorable the Secretary of State transmitting the same to the Honorable the Secretary of the Interior, by whom the paper was referred to me.

The issue of fraudulent diplomas by so-called institutions of learning in our country has been brought in many ways, and often, to the attention of this Office; the institution named in Mr. White's letter is not the only one of this kind known here.

The accompanying data bring out the character of these disgraceful transactions quite unmistakably. After reading them, I trust that you will co-operate in the detection of the offenders and the prevention of a practice so injurious to the credit of learning in the United States, and so opposed to the laws and practices of other nations.

Very respectfully, your obedient servant,

JOHN EATON, *Commissioner.*

*To President of*
BALTIMORE COLLEGE OF DENTAL SURGERY,
Baltimore, Md.

---

[*Mr. Evarts to Mr. Schurz.*]

DEPARTMENT OF STATE,
*Washington, March* 12, 1880.

SIR: I have the honor to transmit herewith for your information a copy of a dispatch (No. 87) of the 2d ultimo, from Mr. White, the Minister of the United States at Berlin, in relation to spurious diplomas issued by a so called American University at Philadelphia. I beg to express the hope that it will be found practicable to devise measures, through the Bureau of Education or otherwise, for the effectual suppression of the practice of issuing spurious diplomas at Philadelphia, which is proving so injurious to the reputation of this country with respect to higher education.

I have the honor to be, sir, your obedient servant,

WM. M. EVARTS.

The Honorable CARL SCHURZ,

*Secretary of the Interior.*

---

[*Mr. White to Mr. Evarts.*]

LEGATION OF THE UNITED STATES,
*Berlin, February* 2, 1880.

SIR: I regret to state that there seems to be a revival here of the sale of diplomas purporting to be issued by an institution of learning in the United States.

Some weeks since a Mr. Pappenheim brought me a diploma, engrossed on parchment in very handsome style, and issued nominally by "the American University at Philadelphia," conferring the degree of doctor of medicine upon one Christopher Schuetz, living, as I understand it, at Leipzig. It would appear that the diploma was offered to Schuetz upon condition of his paying a sum of money for it. It bears the signatures of a number of persons claiming to be professors in the aforesaid University, at the head of them being the signature of "John Buchanan, M. D." Schuetz desired the Legation to give him a declaration of its genuineness and value, which I refused to do. One peculiar feature of the diploma was that, although evidently entirely new and recently issued, it was dated 1872.

About ten days since another and more serious case was brought to my notice. The judicial authorities at Prenzlau forwarded a copy (which I inclose) of a diploma issued by the same alleged institution to Paul Christoph Erdmann Volland, and signed by a faculty at the head of which appears the same name of "John Buchanan, M. D." The authorities at Prenzlau asked the Legation regarding the genuineness of the diploma and the standing of the institution, it being with them a question whether Volland could be allowed to practise his profession under such a diploma.

After looking through the correspondence on record in this Legation (a memorandum of which is inclosed,) and seeking in vain for the name of the institution in the list of colleges and universities published by the Bureau of Education in the Department of the Interior, at Washington, my answer was unfavorable to Volland's claim.

From the correspondence above referred to, I find that attempts have been made by the legislature of Pennsylvania for the suppression of this nuisance; but that, after all, it is a question whether these attempts have been successful, and whether the institution has not still a legal existence. This being the case, I would respectfully suggest that the matter be brought to the notice of the Commissioner of

Education in the Department of the Interior, at Washington, and that he forward me any documents or information in his possession regarding the subject.

You will observe among the papers accompanying the diploma of Volland something much more serious than the diploma itself, and that is the authentication of it by Philip A. Cregar or Gregar, notary public of Philadelphia; and I bring this matter especially to the notice of the Department hoping that something may be done to prevent officials in Pennsylvania lending themselves to what is undoubtedly a fraud, whether under the forms of law or not.

That such cases as these have brought disgrace upon the American system of advanced education and upon the American name in general is certain. This has been recently revealed to me incidentally in a curious way: in a very successful play now running at the Royal Theater in this city, a play written, strangely enough, by a judge of one of the highest tribunals in the Empire, one of the characters, in casting a reflection upon another who is dignified with the title of doctor, declares a belief that the latter had simply bought his degree in America; and in a recent novel, by a popular author here, the scoundrel of the book, having escaped justice in Germany, goes to America, and is at last advices very comfortably settled and practising medicine with a sham diploma which he has bought for money.

All this, of course, is of no especial significance in this case, save as it shows that the fair fame of our country has been and can be injured in the minds of a large number of people even by such contemptible transactions as those herein referred to.

I have the honor to be, sir, your obedient servant,

AND. D. WHITE.

The Hon. WILLIAM M. EVARTS,
*Secretary of State, &c.*

*The Diploma of Volland.**

Omnibus ad quos literæ præsentes pervenerint, præses, curatores professoresque Universitatis Americanæ Philadelphiæ, Reipublicæ Pennsylvaniæ legibus constitutæ, salutem.

Quum in omnibus academiis rite legitimeque constitutis, aut hic aut ubique gentium, usus laudabilis et antiquus fuerit, ut viri, qui vel literis vel artibus ingenuis, vel quibuslibet studiis liberalibus, non minus diligenter quam feliciter operam dederunt, interea recte atque honeste se gerentes, aliquo eximio honore adornarentur, et ad meritam dignitatem attollerentur, et quum nos, secundum leges reipublicæ nostræ, amplissimam potestatem insigniendi decorandique titulis academicis, et promovendi ad gradus in sacra theologia, legibus, artibus liberalibus ac medicina viros bene merentes teneamus, nos igitur, hac auctoritate præditi, usûsque antiqui haud immemores, decrevimus virum egregium, studiis optimus deditum, *Paul Christoph Erdmann Volland,* de cujus eruditione in *chirurgia dentaria arte* et probis moribus satis compertum exploratumque habemus, dignum atque idoneum qui honoretur, ut *vir doctus* altissimo dignitatis gradu; quare uno animo et creavimus et fecimus eum *chirurgiæ dentariæ doctorem,* eique omnia jura et privilegia quæ ad illum gradum attinent dedimus et concessimus.

In quorem fidem, has literas signo magno universitatis literariæ nostræ communiri jussimus, hoc *decimoquarto* die mensis *Octoberis* annoque Domini nostri millesimo octingentesimo *septuagesimo nono.*

JOHN BUCHANAN, M. D.

JOHN J. FULMER, M. D.

ROBT. DEBEUST, M. D.

RICHARD FORBER, M. D.

CHARLES G. POLK, M. D.

C. H. KEHNROTH, M. D.

JAMES COCHRAN, M. D.

J. K. BOWERS, M. D.

SEAL: { Eclectic Medical College and American University, Philadelphia, 1850. }

A. P. BISSELL, LL. D.
JAMES ROBINSON.

---

[*The diploma as given here is an exact copy of the original; the words written in the blank form are indicated by the use of italics.]

*The Notary's Certificate.*

I, Philip A. Cregar, a notary public for the Commonwealth of Pennsylvania residing in the city of Philadelphia, do hereby certify that the diploma hereto annexed from the "American University of Philadelphia" is the regular diploma of that Institution; that the university is a regularly incorporated institution in good standing, and that the signatures on said diploma are genuine and were acknowledged before me in due form of law.

Witness my hand and notarial seal this fourteenth day of October, A. D. 1879.

[SEAL.] PHILIP A. CREGAR,
*Notary Public.*

---

*Certificate of the Prothonotary.*

STATE OF PENNSYLVANIA,
*County of Philadelphia.* } *ss:*

I, William B. Mann, prothonotary of the courts of common pleas of the county of Philadelphia, do hereby certify that Philip A. Cregar, esquire, by whom the annexed certificate was made, was at the time of so doing, and now is, a notary public in and for said county, duly authorized to take acknowledgments and administer oaths, &c., and that I am well acquainted with the handwriting of the said Philip A. Cregar, notary public, and verily believe the signature thereto is genuine.

In witness whereof I have hereunto set my hand and affixed the seal of the said courts this 16th day of October, 1879.

[SEAL.] WILLIAM B. MANN,
*Prothonotary.*

